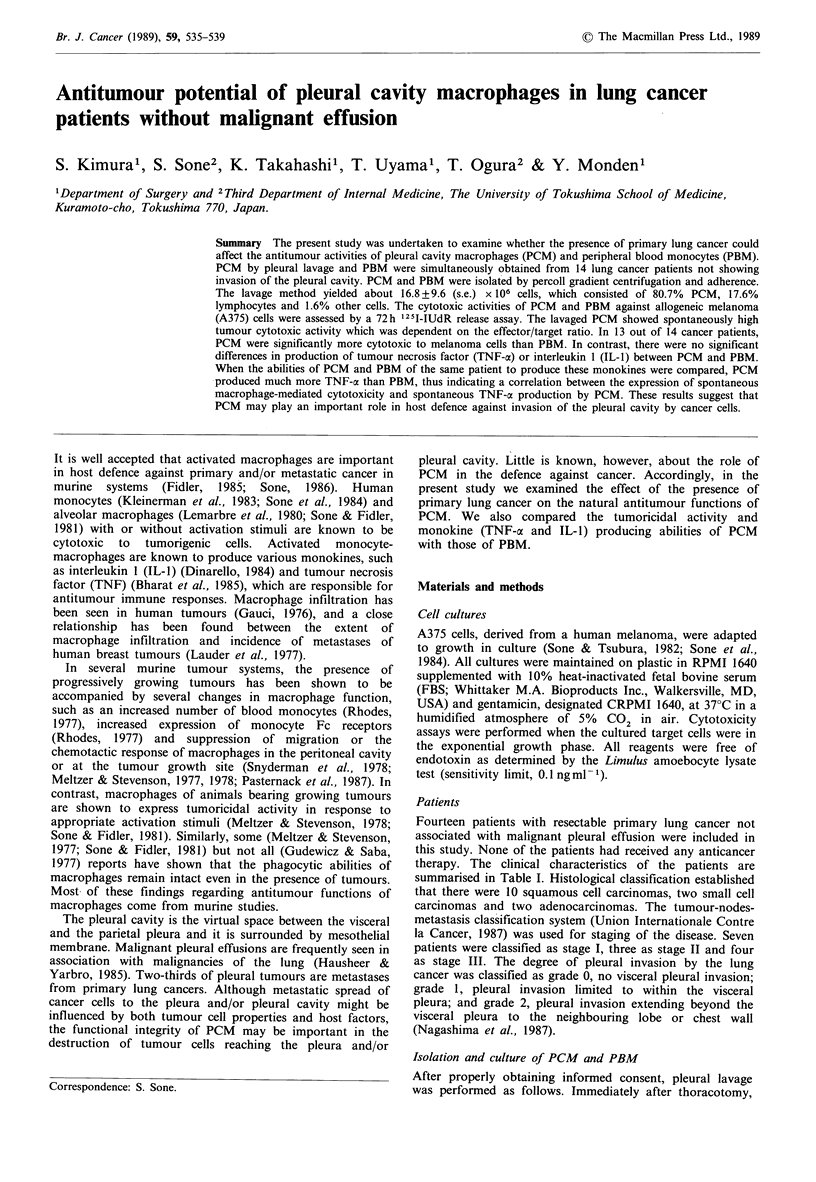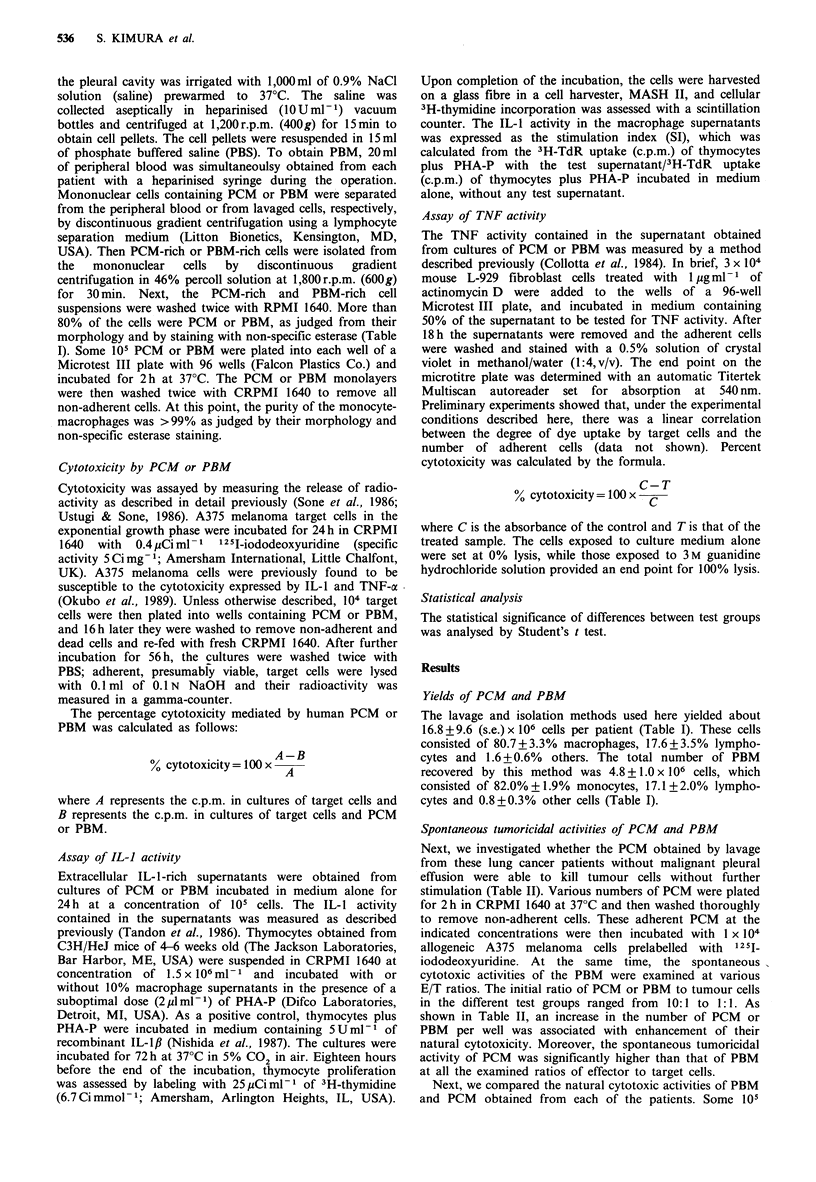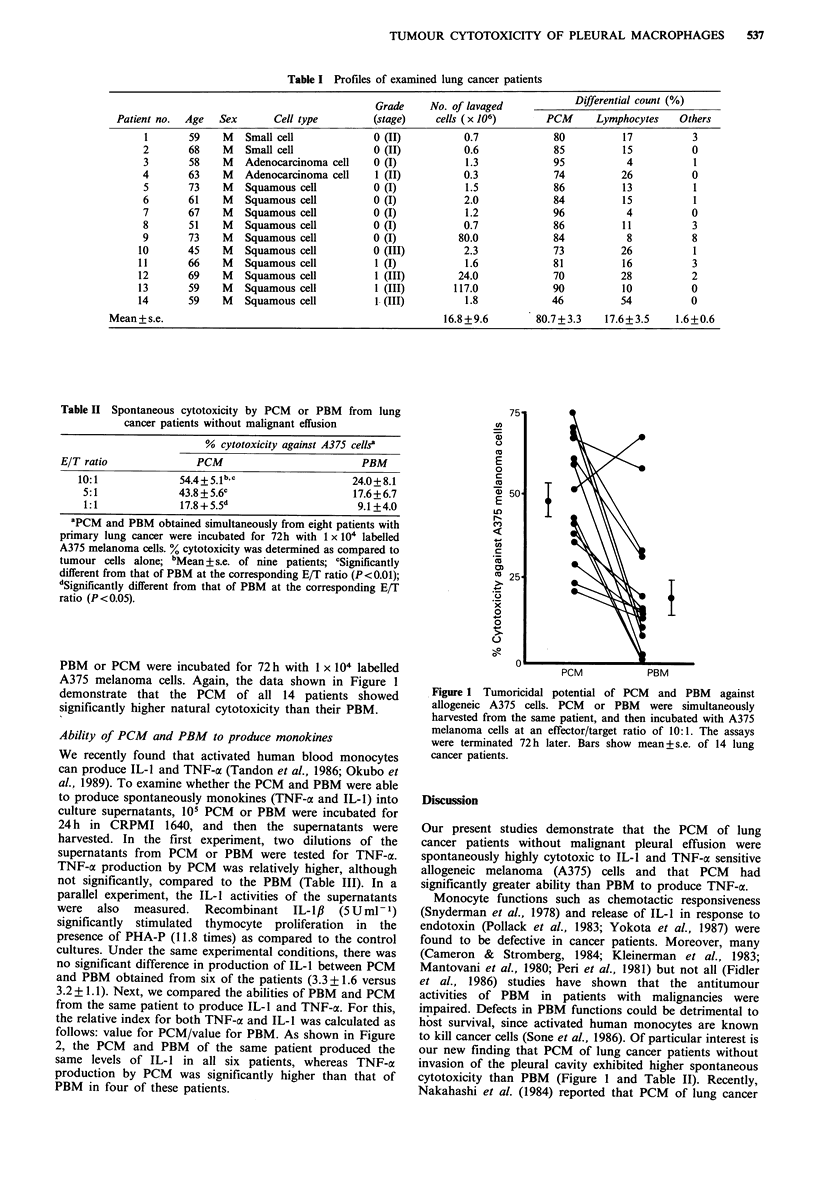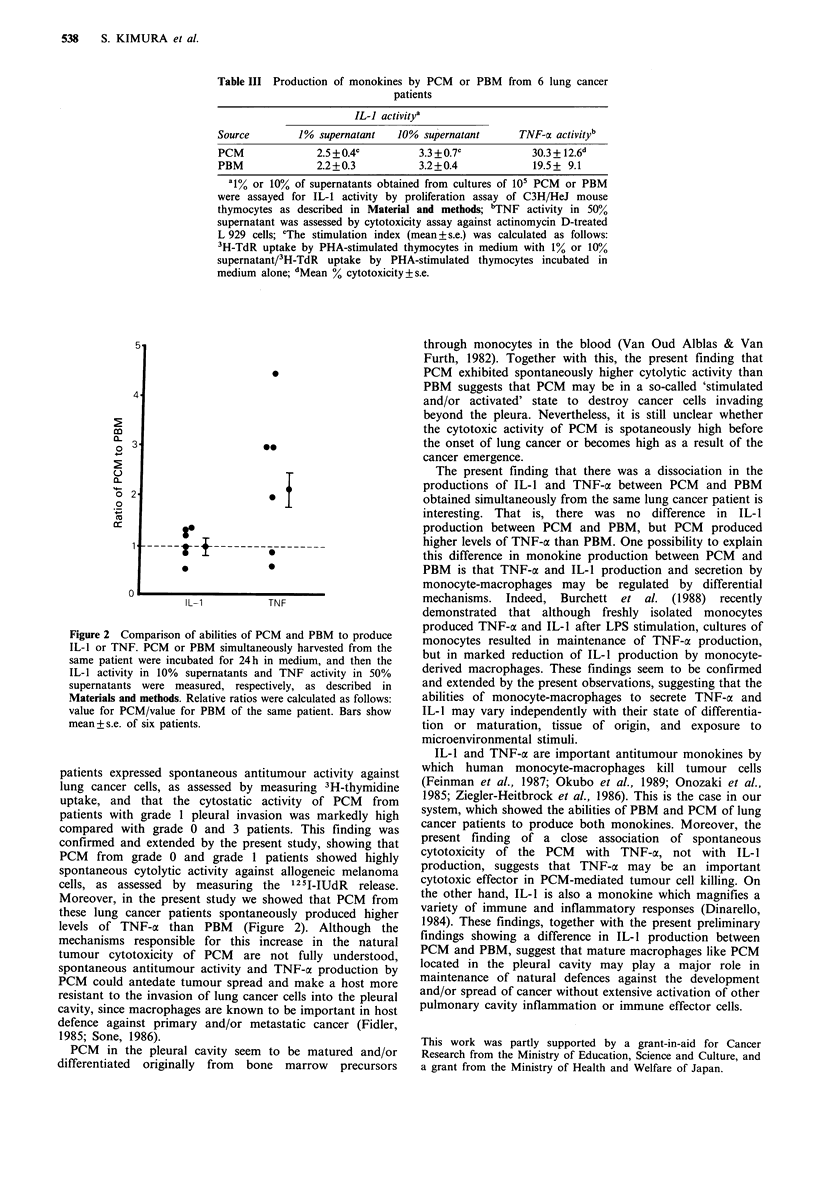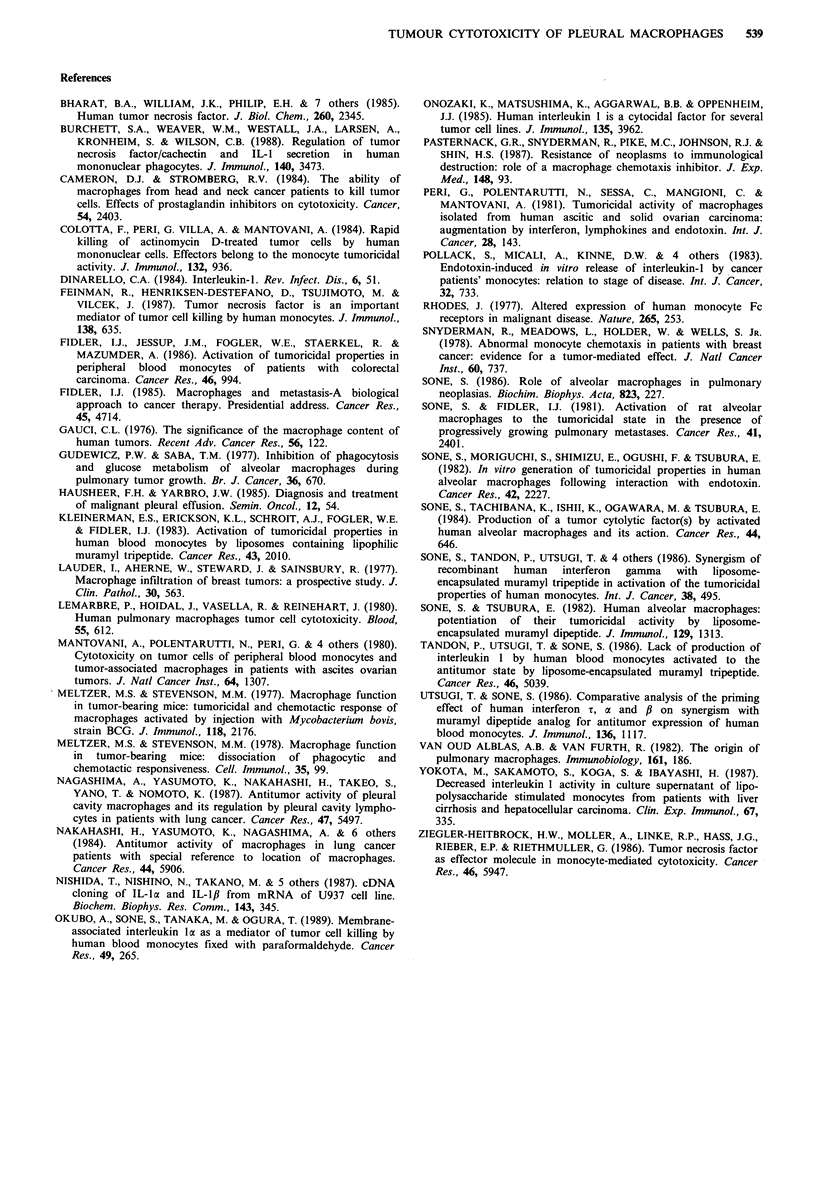# Antitumour potential of pleural cavity macrophages in lung cancer patients without malignant effusion.

**DOI:** 10.1038/bjc.1989.109

**Published:** 1989-04

**Authors:** S. Kimura, S. Sone, K. Takahashi, T. Uyama, T. Ogura, Y. Monden

**Affiliations:** Department of Surgery, University of Tokushima School of Medicine, Japan.

## Abstract

The present study was undertaken to examine whether the presence of primary lung cancer could affect the antitumour activities of pleural cavity macrophages (PCM) and peripheral blood monocytes (PBM). PCM by pleural lavage and PBM were simultaneously obtained from 14 lung cancer patients not showing invasion of the pleural cavity. PCM and PBM were isolated by percoll gradient centrifugation and adherence. The lavage method yielded about 16.8 +/- 9.6 (s.e.) x 10(6) cells, which consisted of 80.7% PCM, 17.6% lymphocytes and 1.6% other cells. The cytotoxic activities of PCM and PBM against allogeneic melanoma (A375) cells were assessed by a 72h 125I-IUdR release assay. The lavaged PCM showed spontaneously high tumour cytotoxic activity which was dependent on the effector/target ratio. In 13 out of 14 cancer patients, PCM were significantly more cytotoxic to melanoma cells than PBM. In contrast, there were no significant differences in production of tumour necrosis factor (TNF-alpha) or interleukin 1 (IL-1) between PCM and PBM. When the abilities of PCM and PBM of the same patient to produce these monokines were compared, PCM produced much more TNF-alpha than PBM, thus indicating a correlation between the expression of spontaneous macrophage-mediated cytotoxicity and spontaneous TNF-alpha production by PCM. These results suggest that PCM may play an important role in host defence against invasion of the pleural cavity by cancer cells.


Br. J. Cancer (1989), 59, 535-539 © The Macmillan Press Ltd., 1989

### Antitumour potential of pleural cavity macrophages in lung cancer patients without malignant effusion

S. Kimura[1], S. Sone[2], K. Takahashi[1], T. Uyama[1], T. Ogura[2] & Y. Monden[1]

[1]*Department of Surgery and* [2]*Third Department of Internal Medicine, The University of Tokushima School of Medicine, Kuramoto-cho, Tokushima 770, Japan.*

**Summary** The present study was undertaken to examine whether the presence of primary lung cancer could affect the antitumour activities of pleural cavity macrophages (PCM) and peripheral blood monocytes (PBM). PCM by pleural lavage and PBM were simultaneously obtained from 14 lung cancer patients not showing invasion of the pleural cavity. PCM and PBM were isolated by percoll gradient centrifugation and adherence. The lavage method yielded about $16.8 \pm 9.6$ (s.e.) $\times 10^6$ cells, which consisted of 80.7% PCM, 17.6% lymphocytes and 1.6% other cells. The cytotoxic activities of PCM and PBM against allogeneic melanoma (A375) cells were assessed by a 72 h $^{125}$I-IUdR release assay. The lavaged PCM showed spontaneously high tumour cytotoxic activity which was dependent on the effector/target ratio. In 13 out of 14 cancer patients, PCM were significantly more cytotoxic to melanoma cells than PBM. In contrast, there were no significant differences in production of tumour necrosis factor (TNF-α) or interleukin 1 (IL-1) between PCM and PBM. When the abilities of PCM and PBM of the same patient to produce these monokines were compared, PCM produced much more TNF-α than PBM, thus indicating a correlation between the expression of spontaneous macrophage-mediated cytotoxicity and spontaneous TNF-α production by PCM. These results suggest that PCM may play an important role in host defence against invasion of the pleural cavity by cancer cells.

It is well accepted that activated macrophages are important in host defence against primary and/or metastatic cancer in murine systems (Fidler, 1985; Sone, 1986). Human monocytes (Kleinerman *et al.*, 1983; Sone *et al.*, 1984) and alveolar macrophages (Lemarbre *et al.*, 1980; Sone & Fidler, 1981) with or without activation stimuli are known to be cytotoxic to tumorigenic cells. Activated monocyte-macrophages are known to produce various monokines, such as interleukin 1 (IL-1) (Dinarello, 1984) and tumour necrosis factor (TNF) (Bharat *et al.*, 1985), which are responsible for antitumour immune responses. Macrophage infiltration has been seen in human tumours (Gauci, 1976), and a close relationship has been found between the extent of macrophage infiltration and incidence of metastases of human breast tumours (Lauder *et al.*, 1977).

In several murine tumour systems, the presence of progressively growing tumours has been shown to be accompanied by several changes in macrophage function, such as an increased number of blood monocytes (Rhodes, 1977), increased expression of monocyte Fc receptors (Rhodes, 1977) and suppression of migration or the chemotactic response of macrophages in the peritoneal cavity or at the tumour growth site (Snyderman *et al.*, 1978; Meltzer & Stevenson, 1977, 1978; Pasternack *et al.*, 1987). In contrast, macrophages of animals bearing growing tumours are shown to express tumoricidal activity in response to appropriate activation stimuli (Meltzer & Stevenson, 1978; Sone & Fidler, 1981). Similarly, some (Meltzer & Stevenson, 1977; Sone & Fidler, 1981) but not all (Gudewicz & Saba, 1977) reports have shown that the phagocytic abilities of macrophages remain intact even in the presence of tumours. Most of these findings regarding antitumour functions of macrophages come from murine studies.

The pleural cavity is the virtual space between the visceral and the parietal pleura and it is surrounded by mesothelial membrane. Malignant pleural effusions are frequently seen in association with malignancies of the lung (Hausheer & Yarbro, 1985). Two-thirds of pleural tumours are metastases from primary lung cancers. Although metastatic spread of cancer cells to the pleura and/or pleural cavity might be influenced by both tumour cell properties and host factors, the functional integrity of PCM may be important in the destruction of tumour cells reaching the pleura and/or pleural cavity. Little is known, however, about the role of PCM in the defence against cancer. Accordingly, in the present study we examined the effect of the presence of primary lung cancer on the natural antitumour functions of PCM. We also compared the tumoricidal activity and monokine (TNF-α and IL-1) producing abilities of PCM with those of PBM.

Correspondence: S. Sone.

### Materials and methods

### Cell cultures

A375 cells, derived from a human melanoma, were adapted to growth in culture (Sone & Tsubura, 1982; Sone *et al.*, 1984). All cultures were maintained on plastic in RPMI 1640 supplemented with 10% heat-inactivated fetal bovine serum (FBS; Whittaker M.A. Bioproducts Inc., Walkersville, MD, USA) and gentamicin, designated CRPMI 1640, at 37°C in a humidified atmosphere of 5% $CO_2$ in air. Cytotoxicity assays were performed when the cultured target cells were in the exponential growth phase. All reagents were free of endotoxin as determined by the *Limulus* amoebocyte lysate test (sensitivity limit, 0.1 ng ml$^{-1}$).

### Patients

Fourteen patients with resectable primary lung cancer not associated with malignant pleural effusion were included in this study. None of the patients had received any anticancer therapy. The clinical characteristics of the patients are summarised in Table I. Histological classification established that there were 10 squamous cell carcinomas, two small cell carcinomas and two adenocarcinomas. The tumour-nodes-metastasis classification system (Union Internationale Contre la Cancer, 1987) was used for staging of the disease. Seven patients were classified as stage I, three as stage II and four as stage III. The degree of pleural invasion by the lung cancer was classified as grade 0, no visceral pleural invasion; grade 1, pleural invasion limited to within the visceral pleura; and grade 2, pleural invasion extending beyond the visceral pleura to the neighbouring lobe or chest wall (Nagashima *et al.*, 1987).

### Isolation and culture of PCM and PBM

After properly obtaining informed consent, pleural lavage was performed as follows. Immediately after thoracotomy, the pleural cavity was irrigated with 1,000 ml of 0.9% NaCl solution (saline) prewarmed to 37°C. The saline was collected aseptically in heparinised (10 U ml$^{-1}$) vacuum bottles and centrifuged at 1,200 r.p.m. (400 *g*) for 15 min to obtain cell pellets. The cell pellets were resuspended in 15 ml of phosphate buffered saline (PBS). To obtain PBM, 20 ml of peripheral blood was simultaneoulsy obtained from each patient with a heparinised syringe during the operation. Mononuclear cells containing PCM or PBM were separated from the peripheral blood or from lavaged cells, respectively, by discontinuous gradient centrifugation using a lymphocyte separation medium (Litton Bionetics, Kensington, MD, USA). Then PCM-rich or PBM-rich cells were isolated from the mononuclear cells by discontinuous gradient centrifugation in 46% percoll solution at 1,800 r.p.m. (600 *g*) for 30 min. Next, the PCM-rich and PBM-rich cell suspensions were washed twice with RPMI 1640. More than 80% of the cells were PCM or PBM, as judged from their morphology and by staining with non-specific esterase (Table I). Some $10^5$ PCM or PBM were plated into each well of a Microtest III plate with 96 wells (Falcon Plastics Co.) and incubated for 2 h at 37°C. The PCM or PBM monolayers were then washed twice with CRPMI 1640 to remove all non-adherent cells. At this point, the purity of the monocyte-macrophages was >99% as judged by their morphology and non-specific esterase staining.

### *Cytotoxicity by PCM or PBM*

Cytotoxicity was assayed by measuring the release of radioactivity as described in detail previously (Sone *et al.*, 1986; Ustugi & Sone, 1986). A375 melanoma target cells in the exponential growth phase were incubated for 24 h in CRPMI 1640 with 0.4 $\mu$Ci ml$^{-1}$ $^{125}$I-iododeoxyuridine (specific activity 5 Ci mg$^{-1}$; Amersham International, Little Chalfont, UK). A375 melanoma cells were previously found to be susceptible to the cytotoxicity expressed by IL-1 and TNF-$\alpha$ (Okubo *et al.*, 1989). Unless otherwise described, $10^4$ target cells were then plated into wells containing PCM or PBM, and 16 h later they were washed to remove non-adherent and dead cells and re-fed with fresh CRPMI 1640. After further incubation for 56 h, the cultures were washed twice with PBS; adherent, presumably viable, target cells were lysed with 0.1 ml of 0.1 N NaOH and their radioactivity was measured in a gamma-counter.

The percentage cytotoxicity mediated by human PCM or PBM was calculated as follows:

$$\%\ \text{cytotoxicity} = 100 \times \frac{A-B}{A}$$

where *A* represents the c.p.m. in cultures of target cells and *B* represents the c.p.m. in cultures of target cells and PCM or PBM.

### *Assay of IL-1 activity*

Extracellular IL-1-rich supernatants were obtained from cultures of PCM or PBM incubated in medium alone for 24 h at a concentration of $10^5$ cells. The IL-1 activity contained in the supernatants was measured as described previously (Tandon *et al.*, 1986). Thymocytes obtained from C3H/HeJ mice of 4–6 weeks old (The Jackson Laboratories, Bar Harbor, ME, USA) were suspended in CRPMI 1640 at concentration of $1.5 \times 10^6$ ml$^{-1}$ and incubated with or without 10% macrophage supernatants in the presence of a suboptimal dose (2 $\mu$l ml$^{-1}$) of PHA-P (Difco Laboratories, Detroit, MI, USA). As a positive control, thymocytes plus PHA-P were incubated in medium containing 5 U ml$^{-1}$ of recombinant IL-1$\beta$ (Nishida *et al.*, 1987). The cultures were incubated for 72 h at 37°C in 5% $CO_2$ in air. Eighteen hours before the end of the incubation, thymocyte proliferation was assessed by labeling with 25 $\mu$Ci ml$^{-1}$ of $^3$H-thymidine (6.7 Ci mmol$^{-1}$; Amersham, Arlington Heights, IL, USA). Upon completion of the incubation, the cells were harvested on a glass fibre in a cell harvester, MASH II, and cellular $^3$H-thymidine incorporation was assessed with a scintillation counter. The IL-1 activity in the macrophage supernatants was expressed as the stimulation index (SI), which was calculated from the $^3$H-TdR uptake (c.p.m.) of thymocytes plus PHA-P with the test supernatant/$^3$H-TdR uptake (c.p.m.) of thymocytes plus PHA-P incubated in medium alone, without any test supernatant.

### *Assay of TNF activity*

The TNF activity contained in the supernatant obtained from cultures of PCM or PBM was measured by a method described previously (Collotta *et al.*, 1984). In brief, $3 \times 10^4$ mouse L-929 fibroblast cells treated with 1 $\mu$g ml$^{-1}$ of actinomycin D were added to the wells of a 96-well Microtest III plate, and incubated in medium containing 50% of the supernatant to be tested for TNF activity. After 18 h the supernatants were removed and the adherent cells were washed and stained with a 0.5% solution of crystal violet in methanol/water (1:4, v/v). The end point on the microtitre plate was determined with an automatic Titertek Multiscan autoreader set for absorption at 540 nm. Preliminary experiments showed that, under the experimental conditions described here, there was a linear correlation between the degree of dye uptake by target cells and the number of adherent cells (data not shown). Percent cytotoxicity was calculated by the formula.

$$\%\ \text{cytotoxicity} = 100 \times \frac{C-T}{C}$$

where *C* is the absorbance of the control and *T* is that of the treated sample. The cells exposed to culture medium alone were set at 0% lysis, while those exposed to 3 M guanidine hydrochloride solution provided an end point for 100% lysis.

### *Statistical analysis*

The statistical significance of differences between test groups was analysed by Student's *t* test.

### Results

### *Yields of PCM and PBM*

The lavage and isolation methods used here yielded about $16.8 \pm 9.6$ (s.e.) $\times 10^6$ cells per patient (Table I). These cells consisted of $80.7 \pm 3.3\%$ macrophages, $17.6 \pm 3.5\%$ lymphocytes and $1.6 \pm 0.6\%$ others. The total number of PBM recovered by this method was $4.8 \pm 1.0 \times 10^6$ cells, which consisted of $82.0\% \pm 1.9\%$ monocytes, $17.1 \pm 2.0\%$ lymphocytes and $0.8 \pm 0.3\%$ other cells (Table I).

### *Spontaneous tumoricidal activities of PCM and PBM*

Next, we investigated whether the PCM obtained by lavage from these lung cancer patients without malignant pleural effusion were able to kill tumour cells without further stimulation (Table II). Various numbers of PCM were plated for 2 h in CRPMI 1640 at 37°C and then washed thoroughly to remove non-adherent cells. These adherent PCM at the indicated concentrations were then incubated with $1 \times 10^4$ allogeneic A375 melanoma cells prelabelled with $^{125}$I-iododeoxyuridine. At the same time, the spontaneous cytotoxic activities of the PBM were examined at various E/T ratios. The initial ratio of PCM or PBM to tumour cells in the different test groups ranged from 10:1 to 1:1. As shown in Table II, an increase in the number of PCM or PBM per well was associated with enhancement of their natural cytotoxicity. Moreover, the spontaneous tumoricidal activity of PCM was significantly higher than that of PBM at all the examined ratios of effector to target cells.

Next, we compared the natural cytotoxic activities of PBM and PCM obtained from each of the patients. Some $10^5$

**Table I** Profiles of examined lung cancer patients

| Patient no. | Age | Sex | Cell type | Grade (stage) | No. of lavaged cells ($\times 10^6$) | Differential count (%) PCM | Lymphocytes | Others |
|---|---|---|---|---|---|---|---|---|
| 1 | 59 | M | Small cell | 0 (II) | 0.7 | 80 | 17 | 3 |
| 2 | 68 | M | Small cell | 0 (II) | 0.6 | 85 | 15 | 0 |
| 3 | 58 | M | Adenocarcinoma cell | 0 (I) | 1.3 | 95 | 4 | 1 |
| 4 | 63 | M | Adenocarcinoma cell | 1 (II) | 0.3 | 74 | 26 | 0 |
| 5 | 73 | M | Squamous cell | 0 (I) | 1.5 | 86 | 13 | 1 |
| 6 | 61 | M | Squamous cell | 0 (I) | 2.0 | 84 | 15 | 1 |
| 7 | 67 | M | Squamous cell | 0 (I) | 1.2 | 96 | 4 | 0 |
| 8 | 51 | M | Squamous cell | 0 (I) | 0.7 | 86 | 11 | 3 |
| 9 | 73 | M | Squamous cell | 0 (I) | 80.0 | 84 | 8 | 8 |
| 10 | 45 | M | Squamous cell | 0 (III) | 2.3 | 73 | 26 | 1 |
| 11 | 66 | M | Squamous cell | 1 (I) | 1.6 | 81 | 16 | 3 |
| 12 | 69 | M | Squamous cell | 1 (III) | 24.0 | 70 | 28 | 2 |
| 13 | 59 | M | Squamous cell | 1 (III) | 117.0 | 90 | 10 | 0 |
| 14 | 59 | M | Squamous cell | 1 (III) | 1.8 | 46 | 54 | 0 |
| Mean ± s.e. | | | | | 16.8±9.6 | 80.7±3.3 | 17.6±3.5 | 1.6±0.6 |

**Table II** Spontaneous cytotoxicity by PCM or PBM from lung cancer patients without malignant effusion

| E/T ratio | % cytotoxicity against A375 cells[a] PCM | PBM |
|---|---|---|
| 10:1 | 54.4±5.1[b,c] | 24.0±8.1 |
| 5:1 | 43.8±5.6[c] | 17.6±6.7 |
| 1:1 | 17.8+5.5[d] | 9.1±4.0 |

[a]PCM and PBM obtained simultaneously from eight patients with primary lung cancer were incubated for 72h with $1 \times 10^4$ labelled A375 melanoma cells. % cytotoxicity was determined as compared to tumour cells alone; [b]Mean±s.e. of nine patients; [c]Significantly different from that of PBM at the corresponding E/T ratio ($P<0.01$); [d]Significantly different from that of PBM at the corresponding E/T ratio ($P<0.05$).

PBM or PCM were incubated for 72h with $1 \times 10^4$ labelled A375 melanoma cells. Again, the data shown in Figure 1 demonstrate that the PCM of all 14 patients showed significantly higher natural cytotoxicity than their PBM.

*Ability of PCM and PBM to produce monokines*

We recently found that activated human blood monocytes can produce IL-1 and TNF-α (Tandon *et al.*, 1986; Okubo *et al.*, 1989). To examine whether the PCM and PBM were able to produce spontaneously monokines (TNF-α and IL-1) into culture supernatants, $10^5$ PCM or PBM were incubated for 24h in CRPMI 1640, and then the supernatants were harvested. In the first experiment, two dilutions of the supernatants from PCM or PBM were tested for TNF-α. TNF-α production by PCM was relatively higher, although not significantly, compared to the PBM (Table III). In a parallel experiment, the IL-1 activities of the supernatants were also measured. Recombinant IL-1β ($5\,U\,ml^{-1}$) significantly stimulated thymocyte proliferation in the presence of PHA-P (11.8 times) as compared to the control cultures. Under the same experimental conditions, there was no significant difference in production of IL-1 between PCM and PBM obtained from six of the patients (3.3±1.6 versus 3.2±1.1). Next, we compared the abilities of PBM and PCM from the same patient to produce IL-1 and TNF-α. For this, the relative index for both TNF-α and IL-1 was calculated as follows: value for PCM/value for PBM. As shown in Figure 2, the PCM and PBM of the same patient produced the same levels of IL-1 in all six patients, whereas TNF-α production by PCM was significantly higher than that of PBM in four of these patients.

**Figure 1** Tumoricidal potential of PCM and PBM against allogeneic A375 cells. PCM or PBM were simultaneously harvested from the same patient, and then incubated with A375 melanoma cells at an effector/target ratio of 10:1. The assays were terminated 72h later. Bars show mean±s.e. of 14 lung cancer patients.

### Discussion

Our present studies demonstrate that the PCM of lung cancer patients without malignant pleural effusion were spontaneously highly cytotoxic to IL-1 and TNF-α sensitive allogeneic melanoma (A375) cells and that PCM had significantly greater ability than PBM to produce TNF-α.

Monocyte functions such as chemotactic responsiveness (Snyderman *et al.*, 1978) and release of IL-1 in response to endotoxin (Pollack *et al.*, 1983; Yokota *et al.*, 1987) were found to be defective in cancer patients. Moreover, many (Cameron & Stromberg, 1984; Kleinerman *et al.*, 1983; Mantovani *et al.*, 1980; Peri *et al.*, 1981) but not all (Fidler *et al.*, 1986) studies have shown that the antitumour activities of PBM in patients with malignancies were impaired. Defects in PBM functions could be detrimental to host survival, since activated human monocytes are known to kill cancer cells (Sone *et al.*, 1986). Of particular interest is our new finding that PCM of lung cancer patients without invasion of the pleural cavity exhibited higher spontaneous cytotoxicity than PBM (Figure 1 and Table II). Recently, Nakahashi *et al.* (1984) reported that PCM of lung cancer

**Table III** Production of monokines by PCM or PBM from 6 lung cancer patients

| Source | *IL-1 activity*[a] | | |
|---|---|---|---|
| | *1% supernatant* | *10% supernatant* | *TNF-α activity*[b] |
| PCM | 2.5±0.4[c] | 3.3±0.7[c] | 30.3±12.6[d] |
| PBM | 2.2±0.3 | 3.2±0.4 | 19.5± 9.1 |

[a]1% or 10% of supernatants obtained from cultures of $10^5$ PCM or PBM were assayed for IL-1 activity by proliferation assay of C3H/HeJ mouse thymocytes as described in **Material and methods**; [b]TNF activity in 50% supernatant was assessed by cytotoxicity assay against actinomycin D-treated L 929 cells; [c]The stimulation index (mean±s.e.) was calculated as follows: $^3$H-TdR uptake by PHA-stimulated thymocytes in medium with 1% or 10% supernatant/$^3$H-TdR uptake by PHA-stimulated thymocytes incubated in medium alone; [d]Mean % cytotoxicity±s.e.

**Figure 2** Comparison of abilities of PCM and PBM to produce IL-1 or TNF. PCM or PBM simultaneously harvested from the same patient were incubated for 24 h in medium, and then the IL-1 activity in 10% supernatants and TNF activity in 50% supernatants were measured, respectively, as described in **Materials and methods**. Relative ratios were calculated as follows: value for PCM/value for PBM of the same patient. Bars show mean±s.e. of six patients.

patients expressed spontaneous antitumour activity against lung cancer cells, as assessed by measuring $^3$H-thymidine uptake, and that the cytostatic activity of PCM from patients with grade 1 pleural invasion was markedly high compared with grade 0 and 3 patients. This finding was confirmed and extended by the present study, showing that PCM from grade 0 and grade 1 patients showed highly spontaneous cytolytic activity against allogeneic melanoma cells, as assessed by measuring the $^{125}$I-IUdR release. Moreover, in the present study we showed that PCM from these lung cancer patients spontaneously produced higher levels of TNF-α than PBM (Figure 2). Although the mechanisms responsible for this increase in the natural tumour cytotoxicity of PCM are not fully understood, spontaneous antitumour activity and TNF-α production by PCM could antedate tumour spread and make a host more resistant to the invasion of lung cancer cells into the pleural cavity, since macrophages are known to be important in host defence against primary and/or metastatic cancer (Fidler, 1985; Sone, 1986).

PCM in the pleural cavity seem to be matured and/or differentiated originally from bone marrow precursors through monocytes in the blood (Van Oud Alblas & Van Furth, 1982). Together with this, the present finding that PCM exhibited spontaneously higher cytolytic activity than PBM suggests that PCM may be in a so-called 'stimulated and/or activated' state to destroy cancer cells invading beyond the pleura. Nevertheless, it is still unclear whether the cytotoxic activity of PCM is spotaneously high before the onset of lung cancer or becomes high as a result of the cancer emergence.

The present finding that there was a dissociation in the productions of IL-1 and TNF-α between PCM and PBM obtained simultaneously from the same lung cancer patient is interesting. That is, there was no difference in IL-1 production between PCM and PBM, but PCM produced higher levels of TNF-α than PBM. One possibility to explain this difference in monokine production between PCM and PBM is that TNF-α and IL-1 production and secretion by monocyte-macrophages may be regulated by differential mechanisms. Indeed, Burchett *et al.* (1988) recently demonstrated that although freshly isolated monocytes produced TNF-α and IL-1 after LPS stimulation, cultures of monocytes resulted in maintenance of TNF-α production, but in marked reduction of IL-1 production by monocyte-derived macrophages. These findings seem to be confirmed and extended by the present observations, suggesting that the abilities of monocyte-macrophages to secrete TNF-α and IL-1 may vary independently with their state of differentiation or maturation, tissue of origin, and exposure to microenvironmental stimuli.

IL-1 and TNF-α are important antitumour monokines by which human monocyte-macrophages kill tumour cells (Feinman *et al.*, 1987; Okubo *et al.*, 1989; Onozaki *et al.*, 1985; Ziegler-Heitbrock *et al.*, 1986). This is the case in our system, which showed the abilities of PBM and PCM of lung cancer patients to produce both monokines. Moreover, the present finding of a close association of spontaneous cytotoxicity of the PCM with TNF-α, not with IL-1 production, suggests that TNF-α may be an important cytotoxic effector in PCM-mediated tumour cell killing. On the other hand, IL-1 is also a monokine which magnifies a variety of immune and inflammatory responses (Dinarello, 1984). These findings, together with the present preliminary findings showing a difference in IL-1 production between PCM and PBM, suggest that mature macrophages like PCM located in the pleural cavity may play a major role in maintenance of natural defences against the development and/or spread of cancer without extensive activation of other pulmonary cavity inflammation or immune effector cells.

This work was partly supported by a grant-in-aid for Cancer Research from the Ministry of Education, Science and Culture, and a grant from the Ministry of Health and Welfare of Japan.

### References

BHARAT, B.A., WILLIAM, J.K., PHILIP, E.H. & 7 others (1985). Human tumor necrosis factor. *J. Biol. Chem.*, **260**, 2345.

BURCHETT, S.A., WEAVER, W.M., WESTALL, J.A., LARSEN, A., KRONHEIM, S. & WILSON, C.B. (1988). Regulation of tumor necrosis factor/cachectin and IL-1 secretion in human mononuclear phagocytes. *J. Immunol.*, **140**, 3473.

CAMERON, D.J. & STROMBERG, R.V. (1984). The ability of macrophages from head and neck cancer patients to kill tumor cells. Effects of prostaglandin inhibitors on cytotoxicity. *Cancer*, **54**, 2403.

COLOTTA, F., PERI, G. VILLA, A. & MANTOVANI, A. (1984). Rapid killing of actinomycin D-treated tumor cells by human mononuclear cells. Effectors belong to the monocyte tumoricidal activity. *J. Immunol.*, **132**, 936.

DINARELLO, C.A. (1984). Interleukin-1. *Rev. Infect. Dis.*, **6**, 51.

FEINMAN, R., HENRIKSEN-DESTEFANO, D., TSUJIMOTO, M. & VILCEK, J. (1987). Tumor necrosis factor is an important mediator of tumor cell killing by human monocytes. *J. Immunol.*, **138**, 635.

FIDLER, I.J., JESSUP, J.M., FOGLER, W.E., STAERKEL, R. & MAZUMDER, A. (1986). Activation of tumoricidal properties in peripheral blood monocytes of patients with colorectal carcinoma. *Cancer Res.*, **46**, 994.

FIDLER, I.J. (1985). Macrophages and metastasis-A biological approach to cancer therapy. Presidential address. *Cancer Res.*, **45**, 4714.

GAUCI, C.L. (1976). The significance of the macrophage content of human tumors. *Recent Adv. Cancer Res.*, **56**, 122.

GUDEWICZ, P.W. & SABA, T.M. (1977). Inhibition of phagocytosis and glucose metabolism of alveolar macrophages during pulmonary tumor growth. *Br. J. Cancer*, **36**, 670.

HAUSHEER, F.H. & YARBRO, J.W. (1985). Diagnosis and treatment of malignant pleural effusion. *Semin. Oncol.*, **12**, 54.

KLEINERMAN, E.S., ERICKSON, K.L., SCHROIT, A.J., FOGLER, W.E. & FIDLER, I.J. (1983). Activation of tumoricidal properties in human blood monocytes by liposomes containing lipophilic muramyl tripeptide. *Cancer Res.*, **43**, 2010.

LAUDER, I., AHERNE, W., STEWARD, J. & SAINSBURY, R. (1977). Macrophage infiltration of breast tumors: a prospective study. *J. Clin. Pathol.*, **30**, 563.

LEMARBRE, P., HOIDAL, J., VASELLA, R. & REINEHART, J. (1980). Human pulmonary macrophages tumor cell cytotoxicity. *Blood*, **55**, 612.

MANTOVANI, A., POLENTARUTTI, N., PERI, G. & 4 others (1980). Cytotoxicity on tumor cells of peripheral blood monocytes and tumor-associated macrophages in patients with ascites ovarian tumors. *J. Natl Cancer Inst.*, **64**, 1307.

MELTZER, M.S. & STEVENSON, M.M. (1977). Macrophage function in tumor-bearing mice: tumoricidal and chemotactic response of macrophages activated by injection with *Mycobacterium bovis*, strain BCG. *J. Immunol.*, **118**, 2176.

MELTZER, M.S. & STEVENSON, M.M. (1978). Macrophage function in tumor-bearing mice: dissociation of phagocytic and chemotactic responsiveness. *Cell. Immunol.*, **35**, 99.

NAGASHIMA, A., YASUMOTO, K., NAKAHASHI, H., TAKEO, S., YANO, T. & NOMOTO, K. (1987). Antitumor activity of pleural cavity macrophages and its regulation by pleural cavity lymphocytes in patients with lung cancer. *Cancer Res.*, **47**, 5497.

NAKAHASHI, H., YASUMOTO, K., NAGASHIMA, A. & 6 others (1984). Antitumor activity of macrophages in lung cancer patients with special reference to location of macrophages. *Cancer Res.*, **44**, 5906.

NISHIDA, T., NISHINO, N., TAKANO, M. & 5 others (1987). cDNA cloning of IL-1$\alpha$ and IL-1$\beta$ from mRNA of U937 cell line. *Biochem. Biophys. Res. Comm.*, **143**, 345.

OKUBO, A., SONE, S., TANAKA, M. & OGURA, T. (1989). Membrane-associated interleukin 1$\alpha$ as a mediator of tumor cell killing by human blood monocytes fixed with paraformaldehyde. *Cancer Res.*, **49**, 265.

ONOZAKI, K., MATSUSHIMA, K., AGGARWAL, B.B. & OPPENHEIM, J.J. (1985). Human interleukin 1 is a cytocidal factor for several tumor cell lines. *J. Immunol.*, **135**, 3962.

PASTERNACK, G.R., SNYDERMAN, R., PIKE, M.C., JOHNSON, R.J. & SHIN, H.S. (1987). Resistance of neoplasms to immunological destruction: role of a macrophage chemotaxis inhibitor. *J. Exp. Med.*, **148**, 93.

PERI, G., POLENTARUTTI, N., SESSA, C., MANGIONI, C. & MANTOVANI, A. (1981). Tumoricidal activity of macrophages isolated from human ascitic and solid ovarian carcinoma: augmentation by interferon, lymphokines and endotoxin. *Int. J. Cancer*, **28**, 143.

POLLACK, S., MICALI, A., KINNE, D.W. & 4 others (1983). Endotoxin-induced *in vitro* release of interleukin-1 by cancer patients' monocytes: relation to stage of disease. *Int. J. Cancer*, **32**, 733.

RHODES, J. (1977). Altered expression of human monocyte Fc receptors in malignant disease. *Nature*, **265**, 253.

SNYDERMAN, R., MEADOWS, L., HOLDER, W. & WELLS, S. JR. (1978). Abnormal monocyte chemotaxis in patients with breast cancer: evidence for a tumor-mediated effect. *J. Natl Cancer Inst.*, **60**, 737.

SONE, S. (1986). Role of alveolar macrophages in pulmonary neoplasias. *Biochim. Biophys. Acta*, **823**, 227.

SONE, S. & FIDLER, I.J. (1981). Activation of rat alveolar macrophages to the tumoricidal state in the presence of progressively growing pulmonary metastases. *Cancer Res.*, **41**, 2401.

SONE, S., MORIGUCHI, S., SHIMIZU, E., OGUSHI, F. & TSUBURA, E. (1982). *In vitro* generation of tumoricidal properties in human alveolar macrophages following interaction with endotoxin. *Cancer Res.*, **42**, 2227.

SONE, S., TACHIBANA, K., ISHII, K., OGAWARA, M. & TSUBURA, E. (1984). Production of a tumor cytolytic factor(s) by activated human alveolar macrophages and its action. *Cancer Res.*, **44**, 646.

SONE, S., TANDON, P., UTSUGI, T. & 4 others (1986). Synergism of recombinant human interferon gamma with liposome-encapsulated muramyl tripeptide in activation of the tumoricidal properties of human monocytes. *Int. J. Cancer*, **38**, 495.

SONE, S. & TSUBURA, E. (1982). Human alveolar macrophages: potentiation of their tumoricidal activity by liposome-encapsulated muramyl dipeptide. *J. Immunol.*, **129**, 1313.

TANDON, P., UTSUGI, T. & SONE, S. (1986). Lack of production of interleukin 1 by human blood monocytes activated to the antitumor state by liposome-encapsulated muramyl tripeptide. *Cancer Res.*, **46**, 5039.

UTSUGI, T. & SONE, S. (1986). Comparative analysis of the priming effect of human interferon $\tau$, $\alpha$ and $\beta$ on synergism with muramyl dipeptide analog for antitumor expression of human blood monocytes. *J. Immunol.*, **136**, 1117.

VAN OUD ALBLAS, A.B. & VAN FURTH, R. (1982). The origin of pulmonary macrophages. *Immunobiology*, **161**, 186.

YOKOTA, M., SAKAMOTO, S., KOGA, S. & IBAYASHI, H. (1987). Decreased interleukin 1 activity in culture supernatant of lipopolysaccharide stimulated monocytes from patients with liver cirrhosis and hepatocellular carcinoma. *Clin. Exp. Immunol.*, **67**, 335.

ZIEGLER-HEITBROCK, H.W., MOLLER, A., LINKE, R.P., HASS, J.G., RIEBER, E.P. & RIETHMULLER, G. (1986). Tumor necrosis factor as effector molecule in monocyte-mediated cytotoxicity. *Cancer Res.*, **46**, 5947.